# Exploratory randomized phase II trial for optimizing treatment dosage and duration of adjuvant S‐1 plus oxaliplatin in patients with stage III colon cancer: YCOG1402 (SOAP trial)

**DOI:** 10.1002/ags3.12687

**Published:** 2023-04-30

**Authors:** Yusuke Suwa, Jun Watanabe, Hirokazu Suwa, Mayumi Ozawa, Masashi Momiyama, Atsushi Ishibe, Kotaro Nagamine, Shigeru Yamagishi, Mitsuyoshi Ota, Tadao Fukushima, Hitoshi Sekido, Yusuke Saigusa, Itaru Endo

**Affiliations:** ^1^ Department of Surgery, Gastroenterological Center Yokohama City University Medical Center Yokohama Japan; ^2^ Department of Surgery Yokosuka Kyosai Hospital Yokosuka Japan; ^3^ Department of Gastroenterological Surgery Yokohama City University Graduate School of Medicine Yokohama Japan; ^4^ Department of Surgery NTT Medical Center Tokyo Tokyo Japan; ^5^ Department of Surgery Yokosuka City Hospital Yokosuka Japan; ^6^ Department of Surgery Fujisawa City Hospital Fujisawa Japan; ^7^ Department of Surgery Yokohama City Minato Red Cross Hospital Yokohama Japan; ^8^ Department of Surgery Saiseikai Yokohamashi Nanbu Hospital Yokohama Japan; ^9^ Department of Surgery National Hospital Organization Yokohama Medical Center Yokohama Japan; ^10^ Department of Biostatistics Yokohama City University Graduate School of Medicine Yokohama Japan

**Keywords:** adjuvant treatment, and S‐1, colorectal cancer, oxaliplatin, SOX

## Abstract

**Introduction:**

Conventionally, the recommended duration of adjuvant chemotherapy of colon cancer had been 6 months. The IDEA Collaboration suggested that shortening capecitabin and oxaliplatin (CAPOX) adjuvant chemotherapy may be possible. S‐1 and oxaliplatin (SOX) treatment is standard treatment in metastatic colorectal cancer in Japan. The aim of this study was to optimize treatment dosage and duration of adjuvant SOX in stage III colon cancer.

**Methods:**

This trial was as open‐label multi‐center randomized phase II study. Patients with stage III colon cancer were randomly assigned to 3 months or 6 months of adjuvant SOX treatment in different doses: 130 mg/m^2^ (3 months) or 100 mg/m^2^ (6 months) of oxaliplatin. The primary endpoint was 3‐year disease‐free survival (DFS) and the null hypothesis for the primary endpoint was that the 3‐year DFS was ≤72% in each arm and was tested with a one‐sided significance level of 10%.

**Results:**

Eighty‐two patients were assigned to the 6 months arm and 81 to the 3 months arm. The 3‐year DFS was 75.0% (80% CI 67.95–80.72, *p* = 0.282) in the 6 months arm and 76.9% (80% CI 70.1–82.38, *p* = 0.171) in the 3 months arm. Treatment completion rate and relative dose intensity (RDI) were higher in 3 months than 6 months arm. The adverse events (AE) were similar in both arms.

**Conclusions:**

The 3‐year DFS was not significantly superior to null hypothesis in both 3 months and 6 months arms for the stage III colon cancer. Primary endpoint was not achieved. The SOX regimen was not feasible in long‐term outcomes.

## Graphical abstract

S‐1 and oxaliplatin (SOX) adjuvant chemotherapy of 130 mg/m2 (3 months) or 100 mg/m2 (6 months) of oxaliplatin was feasible and safe for the patients with stage III colon cancer.

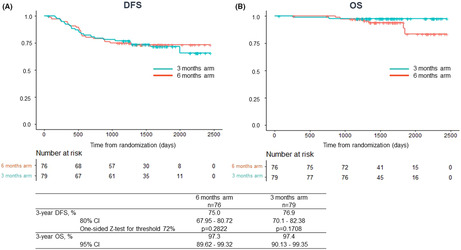

## 1 INTRODUCTION

Conventionally, the recommended duration of adjuvant chemotherapy of colon cancer had been 6 months. The IDEA Collaboration showed that 0.4% difference in 5‐year overall survival between 3‐month and 6‐month of adjuvant treatment with oxaliplatin‐based regimen for the patients with Stage III colon cancer. It did not show the non‐inferiority of 3‐months.^1^ However, this gap should be placed in clinical context, and this study suggested that shortening the oxaliplatin‐based adjuvant chemotherapy may be possible. Although the direct comparison between FOLFOX and CAPOX regimen was not performed in this trial, a longer duration of therapy did not provide additional benefit, with non‐inferiority shown (74·8% vs. 75·9%; hazard ratio [HR] 0·95 [95% CI 0·85–1·06]) in patients treated with CAPOX, whereas 6 months of FOLFOX therapy significantly improved 3‐year DFS compared with that of 3 months of therapy (73·6% vs. 76·0%; 1·16 [1·06–1·26]).^2^ This outcome was associated not only with duration and kind of regimen. CAPOX might be superior to FOLFOX regimen in adjuvant chemotherapy for stage III colon cancer. The ACHIEVE study, one of the IDEA collaborations conducted in Japan, showed that the 3‐year OS in 3 months of CAPOX was equivalent to 6 months in the patient of the IDEA low‐risk stage III (T1‐3 and N1).^3^

S‐1 contains gimeracil, which helps to inhibit the degradation of 5‐FU in the body, and oteracil, which helps to reduce gastrointestinal side effects normally caused by 5‐FU. It is an oral agent that is converted to 5‐FU in the body as well as capecitabin. The S‐1 and oxaliplatin (SOX) regimen was originally investigated in Japan for colon cancer and advanced gastric cancer. And the SOX and SOX plus bevacizumab regimens are recommended in 1st‐line chemotherapy for the patients with metastatic colorectal cancer in Japanese Society for Cancer of the Colon and Rectum guidelines 2022 for the treatment of colorectal cancer,^4^ and Pan‐Asian adapted ESMO consensus guidelines for the management of patients with metastatic colorectal cancer.^5^ Based on these backgrounds, it was necessary and meaningful to develop the adjuvant treatment of SOX regimen in Japan. Moreover, whether the SOX regimen was expected to be one of the options for adjuvant chemotherapy in the future depends on the ACT‐CC 02 trial results at the time this study was planned and initiated, as well as CAPOX regimen, which had to be optimized as an adjuvant therapy in terms of duration and dosage.

The aim of our study is basically an exploratory examination of optimal therapeutic dosage and duration of SOX adjuvant treatment. Therefore, we set oxaliplatin dosage at 6 months at 100 mg/m^2^ and at 3 months at 130 mg/m^2^.

## 2 METHODS

### 2.1 Study design

This trial was as open‐label multi‐center randomized phase II study as YCOG1403 study. This study was conducted in the Yokohama Clinical Oncology Group (YCOG) institutes. The institutional review board at Yokohama City University and each study center approved the protocol in accordance with the principles expressed in the Declaration of Helsinki. All patients provided written informed consent prior to enrollment. This study is registered with the UMIN Clinical Trial Registry (UMIN000013825).

### 2.2 Patients

Patients who had undergone curative resection for stage III colon cancer and enrolled by study investigators were randomized 1:1–6 months and 3 months of adjuvant SOX delivered every 3 weeks in different doses according to stratification factor: primary tumor site (colon or rectosigmoid vs. upper rectum), the number of metastatic lymph nodes (≤3 vs. >3), and institutions.

The inclusion criteria were that an Eastern Cooperative Oncology Group (ECOG) performance status was 0–1, the age was between 20 and 80 years old, and the vital organ function was adequate: white blood cell count ≥3000/mm^3^, neutrophil count ≥1500/mm^3^, platelet count ≥100 000/mm^3^, hemoglobin ≥9.0 g/dL, total bilirubin ≤2.0 mg/dL, aspartate aminotransferase (AST) and alanine aminotransferase (ALT) ≤100 IU/L, creatinine clearance (CCr, calculated using Cockcroft‐Gault equation value) ≥60 mL/min. The exclusion criteria were that the patients had another sever comorbidity, prior chemotherapy or radiation therapy history, history of another cancer in the last 5 years, and the peripheral neuropathy of grade 1 and higher.

### 2.3 Treatment

The treatment was not blinded to patients and investigators. Treatment in SOX included a 2‐hour intravenous infusion of 130 mg/m^2^ (3 months arm) or 100 mg/m^2^ (6 months arm) of oxaliplatin on day 1 and oral S‐1 80 mg/m^2^ according to BSA twice a day from the evening of day 1 to the morning of day 15 repeated every 3 weeks. Four cycles and eight cycles SOX was administered to patients in the 3‐month and 6‐month arms, respectively.

### 2.4 PNQ questionnaire

**FIGURE 1:**
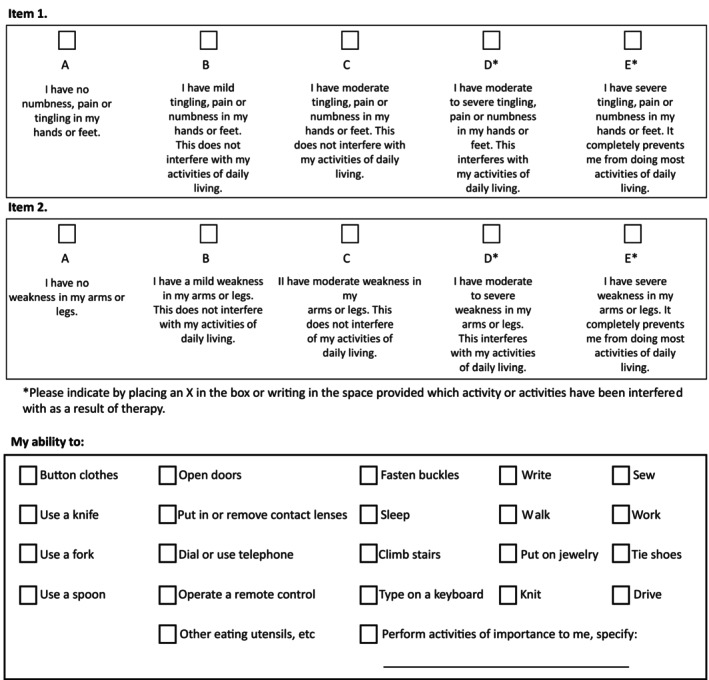
The Peri‐neuropathy Questionnaire (PNQ).^6^

We assessed the chemotherapy‐induced peripheral neuropathy (CIPN) using the Peri‐neuropathy Questionnaire (PNQ)^6^ (Figure 1), which was translated in Japanese, from pretreatment to 12 months after the end of the treatment. It was directly collected pretreatment, before the start of every course, and at 3, 6, 9, and 12 months after the end of adjuvant treatment from participants. We translated A, B, C, D, E in PNQ to 1, 2, 3, 4, 5 for analysis, respectively.

### 2.5 Follow‐up

Patients were evaluated for recurrence with CEA and CA19‐9 value in plasma every 3 months, and chest, abdominal and pelvis computed tomography every 6 months, and colonoscopy every 1 year.

### 2.6 Outcomes

The primary endpoint was 3‐year DFS. The secondary endpoints were OS, RDI, treatment completion rate, and AE. Evaluation of AE was based on the CTCAE ver. 4.0.

### 2.7 Statical analyses

ACTS‐CC, JCOG0205, and JFMC33‐0502 studies conducted in Japan verified 5‐FU‐based adjuvant chemotherapy without oxaliplatin and reported a 3‐year DFS of 72.5%–79.3%.^7^, ^8^, ^9^ Therefore, the threshold 3‐year DFS was set at 72%. MOSAIC and XELOXA (NO16968) trials reported that the additional of oxaliplatin to 5‐FU‐based adjuvant chemotherapy resulted in additional effect to 3‐year DFS of 4.4%–6.9%.^10^, ^11^, ^12^ Based on a 3‐year DFS of 75.5% for the ACTS‐CC S‐1 arm, a 3‐year DFS for SOX therapy was expected to be 82%. Assuming an expected 3‐year DFS of 82%, the significance level *α* = 0.1 (one‐sided) for 78 patients in one arm, and the power of the test for the null hypothesis of 72% was 77%. Considering some ineligible cases, the planned sample size was set at 80 cases per arm. The purpose of the primary analysis of this study was to determine whether the 3‐year DFS of protocol treatment in each arm had efficacy that allowed rejection of the threshold (P0) for each arm.

Efficacy analysis was performed in all registered and randomized patients. Safety analysis was performed in all patients who had received SOX adjuvant chemotherapy. The DFS and OS were estimated with the Kaplan–Meier method and the Greenwood formula. The one‐sample test for 3‐year DFS was performed using the Greenwood formula to estimate the standard deviation of the complementary log–log transformed survival. The Fisher test and t‐test were employed to compare the treatment completion rates and the mean RDI between arms, respectively. All statistical analyses were performed using the SAS software, version 9.4 or later (SAS Institute) or, the R software, version 4.1.0 or later (R Foundation for Statistical Computing, Vienna, Austria).

## 3 RESULTS

### 3.1 Patient characteristics

**FIGURE 2:**
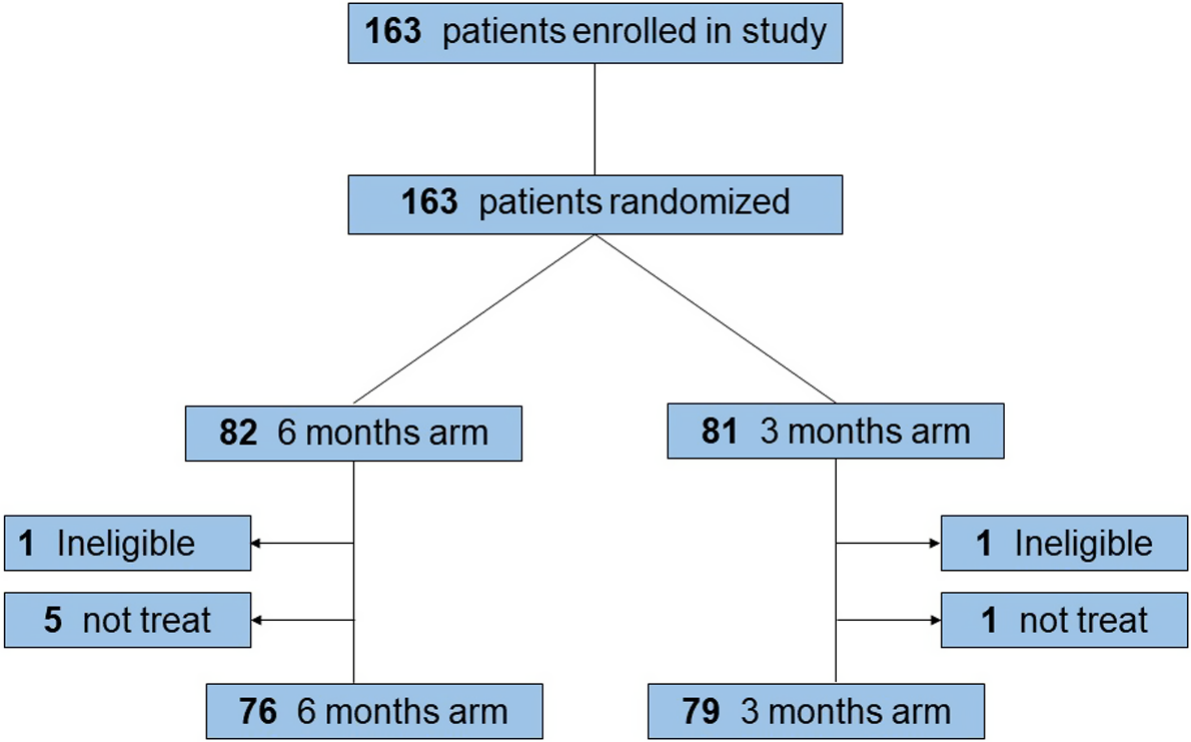
CONSORT Flow Diagram.

Patients were enrolled from nine institutions in Japan that belonged to YCOG and randomly assigned to 6 months or 3 months SOX adjuvant chemotherapy between May 1, 2014, and November 11, 2017. Eighty‐two patients were assigned to the 6 months arm and 81 patients assigned to the 3 months arm. Two cases assigned to the 3 months arm and six cases in 6 months arm were excluded from the primary analyses: four patients in 6 months arm and one patent in 3 months arm could not start protocol treatment because they did not meet starting criteria after randomization, and the mistake in the pathological result were found in one patient in 3 months arm and one patient in 3 months arm after randomization, and the randomization error was found in one case. Finally, the primary analysis was performed in 76 patients in the 6 months and 79 patients in 3 months arm (Figure 2).

There were no substantial differences in the baseline characteristics between the two arms. Eighteen patients (23.6%) in 6 months arm and 20 patients (25.3%) in 3 months arm were pathological T4 and 15 patients (19.7%) and 18 patients (22.8%) were pN2, respectively, and there was no difference in the rate of high‐risk stage III patients between two arms (Table 1).

**TABLE 1:** Patient characteristics.

Characteristic	Treatment arm		
	6 months arm	3 months arm	
	*n* = 76	*n* = 79	*p* value
Age, median (IQR), year	64.5 (58.0–69.5)	65.0 (57.0–70.0)	0.817
Gender, *n* (%)			
Male	49 (64.5)	42 (53.2)	0.192
Female	27 (35.5)	37 (46.8)	
ECOG PS, *n* (%)			
0	73 (96.1)	77 (97.5)	0.677
1	3 (3.9)	2 (2.5)	
Body Mass Index, median (IQR), kg/m^2^	22.04 (19.61–23.39)	21.11 (19.09–23.59)	0.382
Body Surface Area, median (IQR), m^2^	1.60 (1.505–1.715)	1.58 (1.46–1.72)	0.3
Location of tumor, *n* (%)			
Left side	57	61	0.746
Right side	19	18	
Size of tumor, median (IQR), mm	40.0 (27.5–53.5)	40.0 (25.0–50.0)	
T stage, *n* (%)			
pT1	7 (9.2)	10 (12.7)	0.848
pT2	11 (14.5)	7 (8.9)	
pT3	40 (52.6)	42 (53.2)	
pT4	18 (23.6)	20 (25.3)	
N stage, *n* (%)			
N1	61 (80.3)	61 (77.2)	0.697
N2	15 (19.7)	18 (22.8)	
No. of relative lymph node, median (IQR)	22.5 (16.0–34.0)	26.0 (18.0–34.0)	
No. of positive lymph node, median (IQR)	2.0 (1.0–3.0)	2.0 (1.0–3.0)	
pStage, *n* (%)			
IIIA	18 (23.7)	14 (17.7)	0.678
IIIB	48 (63.2)	54 (68.4)	
IIIC	10 (13.2)	11 (13.9)	

Abbreviations: ECOG PS, Eastern Cooperative Oncology Group Performance status; IQR, interquartile range.

### 3.2 The DFS and OS

**FIGURE 3:**
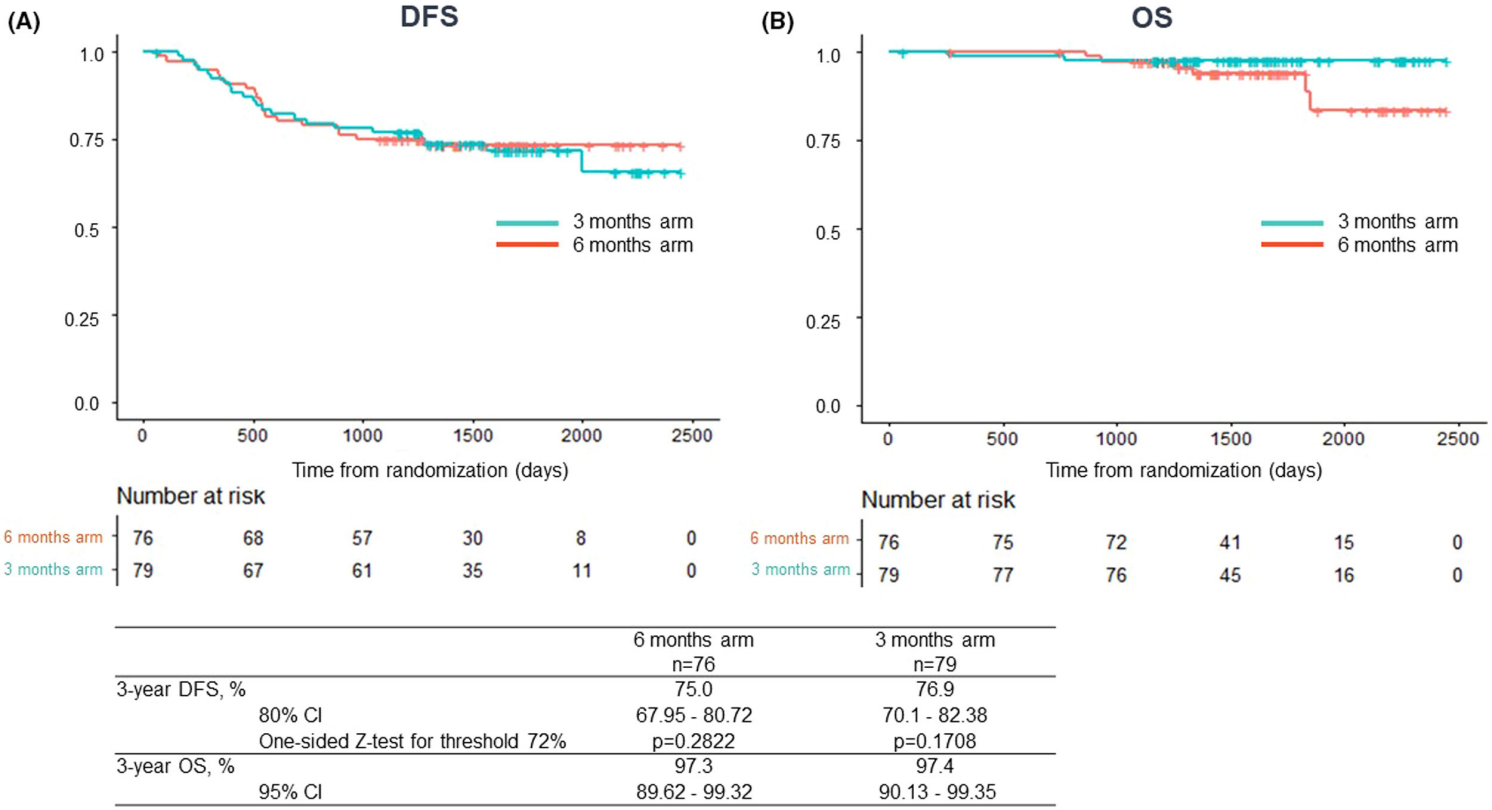
(A) The Kaplan–Meier curve of disease‐free survival in 6 months and 3 months arm. (B) The Kaplan–Meier curve of overall survival in 6 months and 3 months arm.

The median duration of follow‐up after randomization was 52.1 months (IQR 42.8–59.4). The 3‐year DFS was 75.0% (80% CI 67.95–80.72, *p* = 0.282) in the 6 months arm and 76.9% (80% CI 70.1–82.38, *p* = 0.171) in the 3 months arm (Figure 3A). Our outcomes means that the 3‐year DFS of protocol treatment in each arm did not have efficacy that allowed rejection of the threshold (P0) for each arm. The 3‐year OS was 97.3% (95% CI 89.62–99.32) and 97.4% (95% CI 90.13–99.35), respectively (Figure 3B).

### 3.3 The treatment completion and dose intensity

Treatment completion rate was 73.7% in the 6 months arm and 86.1% in the 3 months arm (*p* = 0.070). The mean RDI of oxaliplatin and S‐1 were 76.9% and 77.4% in the 6 months arm and 82.6% and 85.0% in the 3 months arm, respectively (*p* = 0.030, *p* = 0.002). (Table 2).

**TABLE 2:** Relative dose intensity in 6 months and 3 months arms.

	6 months arm	3 months arm	*p* value
RDI: oxaliplatin (%), N	76	79	0.002
mean (SD)	77.4 (15.1)	85.0 (15.1)	
median (IQR)	78.5 (65.9–89.4)	88.5 (73.6–100.0)	
RDI: S‐1 (%), N	76	79	0.03
mean (SD)	76.9 (14.9)	82.6 (17.5)	
median (IQR)	78.6 (65.1–88.9)	85.7 (70.6–100.0)	

Abbreviations: IQR, interquartile range; RDI, Relative Dose Intensity; SD, Standard Deviation.

### 3.4 Safety

The rate of grade 3 and more AE was 42.1% in the 6 months arm and 34.2% in the 3 months arm (*p* = 0.326). The overall and grade 3 or 4 neutrophil count decrease was 61.8% and 19.7% in 6 months arm, and 41.8% and 13.9% in 3 months arm, respectively. Overall neutropenia was greater in 6 months arms than in 3 months arms (Table 3). However, there was no patients with febrile neutropenia both arms.

**TABLE 3:** Adverse events in 6 months and 3 months arm.

	6 months arm, *n* (%) *n* = 76	3 months arm, *n* (%) *n* = 79	*p* Value
Mucositis oral			
All grade	20 (26.3)	24 (30.4)	0.597
Grade 3 and 4	1 (1.3)	0 (0.0)	0.490
FN			
All grade	0 (0.0)	0 (0.0)	1.000
Grade 3 and 4	0 (0.0)	0 (0.0)	1.000
Allergy reaction			
All grade	1 (1.3)	0 (0.0)	0.490
Grade 3 and 4	1 (1.3)	0 (0.0)	0.490
Fatigue			
All grade	14 (18.4)	11 (13.9)	0.515
Grade 3 and 4	4 (5.3)	2 (2.5)	0.436
Malaise			
All grade	44 (57.9)	37 (46.8)	0.199
Grade 3 and 4	0 (0.0)	0 (0.0)	1.000
Anorexia			
All grade	32 (42.1)	35 (44.3)	0.871
Grade 3 and 4	8 (10.5)	3 (3.8)	0.126
Nausea			
All grade	34 (44.7)	32 (40.5)	0.628
Grade 3 and 4	5 (6.6)	2 (2.5)	0.270
Vomiting			
All grade	15 (19.7)	12 (15.2)	0.527
Grade 3 and 4	3 (3.9)	0 (0.0)	0.115
Diarrhea			
All grade	37 (48.6)	36 (45.6)	0.748
Grade 3 and 4	9 (11.8)	11 (13.9)	0.812
Constipation			
All grade	19 (25.0)	27 (34.2)	0.223
Grade 3 and 4	0 (0.0)	0 (0.0)	1.000
Hand‐foot syndrome			
All grade	7 (9.2)	2 (2.5)	0.093
Grade 3 and 4	1 (1.3)	0 (0.0)	0.490
Peripheral sensory neuropathy			
All grade	65 (85.5)	63 (79.7)	0.400
Grade 3 and 4	2 (2.6)	0 (0.0)	0.239
Peripheral motor neuropathy			
All grade	3 (3.9)	4 (5.1)	1.000
Grade 3 and 4	0 (0.0)	0 (0.0)	1.000
White blood cell decreased			
All grade	32 (42.1)	31 (39.2)	0.745
Grade 3 and 4	2 (2.6)	0 (0.0)	0.239
Neutrophil count decreased			
All grade	47 (61.8)	33 (41.8)	0.015
Grade 3 and 4	15 (19.7)	11 (13.9)	0.393
Anemina			
All grade	33 (43.4)	34 (43.0)	1.000
Grade 3 and 4	0 (0.0)	0 (0.0)	1.000
Platelet count decreased			
All grade	51 (67.1)	41 (51.9)	0.071
Grade 3 and 4	1 (1.3)	2 (2.5)	1.000
AST increased			
All grade	38 (50.0)	31 (39.2)	0.198
Grade 3 and 4	1 (1.3)	1 (1.3)	1.000
ALT increased			
All grade	29 (38.2)	24 (31.6)	0.316
Grade 3 and 4	1 (1.3)	1 (1.3)	1.000
Blood bilirubin increased			
All grade	10 (13.2)	4 (5.1)	0.096
Grade 3 and 4	0 (0.0)	0 (0.0)	1.000
Creatinine increased			
All grade	3 (3.9)	1 (1.3)	0.360
Grade 3 and 4	0 (0.0)	0 (0.0)	1.000

Abbreviations: ALT, alanine aminotransferase; AST, aspartate aminotransferase; FN, Febrile Neutropenia.

### 3.5 Chemotherapy‐induced peripheral neuropathy

**FIGURE 4:**
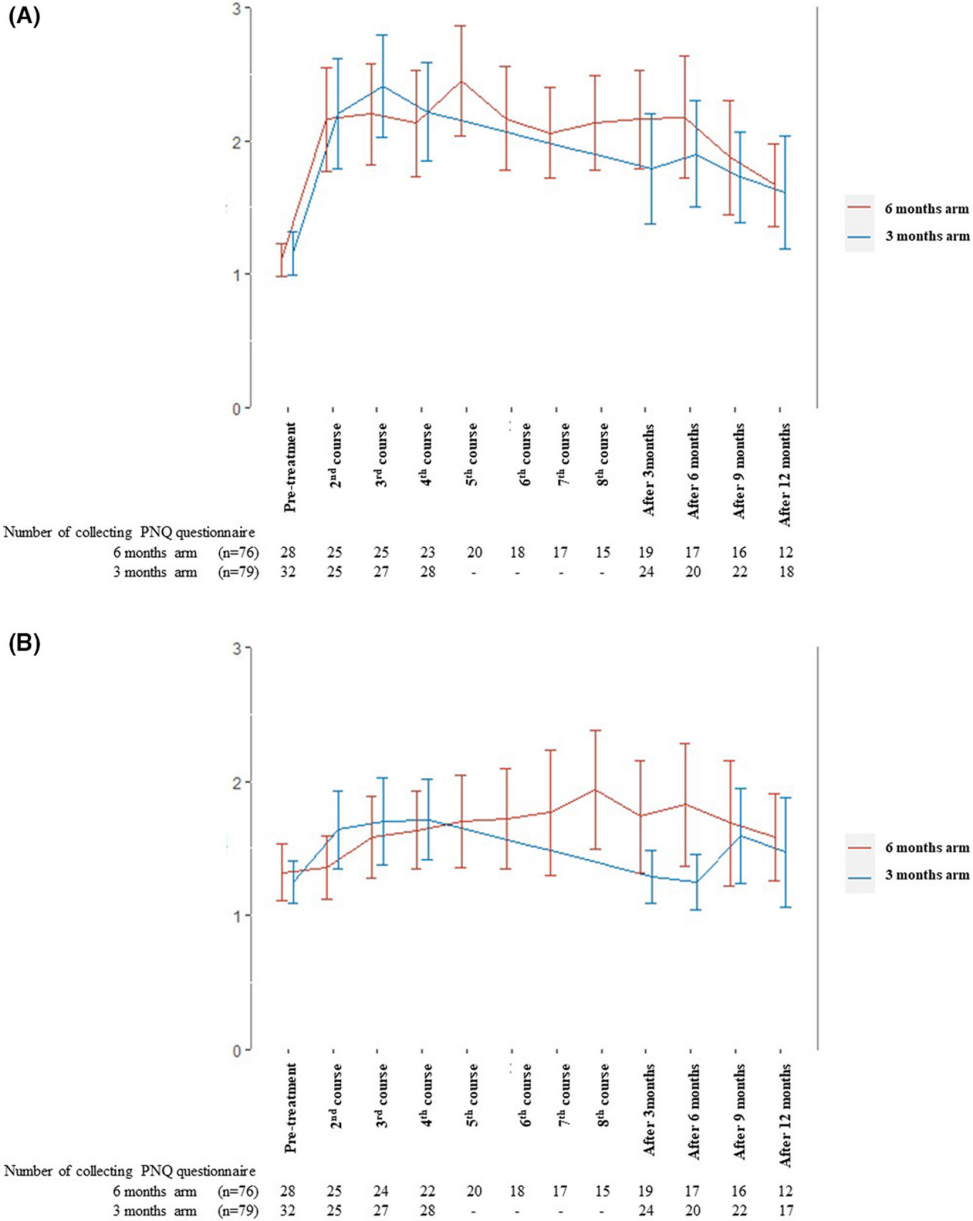
(A) The motor peri‐neuropathy had no difference between 6 months and 3 months arms during treatment and post treatment on PNQ. (B) The sensory peri‐neuropathy had no difference between 3 months and 6 months arms during treatment and post treatment on PNQ.

The rate of any grade CIPN was 85.5% in 6 months arm and 79.7% in 3 months arm, respectively. The incidence of CIPN grade 3 and more was two patients (2.6%) in 6 months arm and there were no patients in 3 months arm (Table 3). The sensory and motor peripheral neuropathy had no difference from two arms both during treatment and post treatment on PNQ. (Figure 4A,B).

## 4 DISCUSSION

This study was the first study to optimize treatment dosage and duration of SOX regimen in patients with stage III colon cancer in adjuvant setting. The adjuvant treatment of stage III colon cancer after curative surgery is standard with oxaliplatin‐based regimen, FOLFOX or CAPOX, were superior to 5‐FU (MOSAIC) NO16968 Randomized Controlled Phase III Trial.^10^, ^11^, ^12^

The SOX regimen was shown to be non‐inferior to CAPOX in terms of PFS as a first‐line treatment for metastatic colorectal cancer in a phase III trial conducted in Korea.^13^ In addition, the SOFT trial showed non‐inferiority of SOX plus bevacizumab to mFOLFOX6 plus bevacizumab as first‐line treatment in a phase III trial in Japan.^14^

In adjuvant treatment for the colon cancer, S‐1 was confirmed to be noninferior in DFS compared with UFT / LV in ACTS‐CC trial. It showed that 3‐year DFS was 72.5% in UFT / LV arm and 75.5% in S‐1 arm (HR = 0.85 (95% CI: 0.70–1.03 *p* < 0.0001)).^7^ However, S‐1 could not show non‐inferiority compared with capecitabine for DFS for stage III colorectal cancer in JCOG0910 trial. It showed that 3‐year DFS was 82.0% (95% CI: 78.5–85.0) in capecitabine arm and 77.9% (95% CI: 74.1–81.1) in S‐1 arm. This result means that S‐1 was not recommended in adjuvant treatment for stage III colorectal cancer.^15^ However, it remains to be seen whether SOX treatment is not simila compared to CAPOX in adjuvant setting.

SOX treatment is one of the standard chemotherapies for the patients with advanced gastric cancer as well as CAPOX treatment. SOX treatment was non‐inferior to S‐1 plus CDDP (SP) treatment in the first‐line chemotherapy for the patients with advanced gastric cancer.^16^ In adjuvant treatment, S‐1 is standard for the patients with stage II or III gastric cancer.^17^, ^18^ The CAPOX regimen after D2 gastrectomy was subsequently shown to be superior compared to observation group in CLASSIC trial in stage II or III gastric cancer,^19^ and it meant that oxaliplatin‐based regimen was effective in adjuvant therapy for gastric cancer. However, the SOX treatment in adjuvant therapy for stage II or III gastric cancer patients has not been evaluated in phase 3 trials yet. However, it is suggested to be manageable and safe in selected patients for stage III gastric cancer in phase 2 study,^20^ and the exploratory analysis of some studies was showed that SOX adjuvant treatment are suggested to have similar efficacy to CAPOX for stage III gastric cancer patients after D2 gastrectomy.^21^ Therefore, doublet platinum (oxaliplatin or cisplatin) and fluoropyrimidine (infusional 5‐FU, S‐1, or Capecitabine) combinations are recommended for fit patients with advanced gastric cancer for the management of patients with metastatic gastric cancer in the Pan‐Asian adapted ESMO Clinical Practice Guidelines.^22^

The adjuvant SOX treatment has not yet been established for colon cancer. SOX treatment was compared with UFT/ LV in adjuvant setting in ACTS‐CC 02 trial,^23^ which was ongoing when our trial was planned and started. This study showed the 3‐year DFS was 62.7% (95% CI, 58.1–66.9) in the SOX group, compared to 60.6% (95% CI, 56.0–64.9) in the UFT/LV group. It was not superior to UFT/LV in patients with high‐risk stage III colon cancer (any T and N2, or any T and positive main lymph nodes) after R0 resection. However, the increase in the 3‐year DFS rate in the SOX group as compared with the UFT/LV group in stage IIIC, T4, and N2b patients was 5.2% (HR, 0.82; 95% CI 0.63–1.06), 4.2% (HR, 0.85; 95% CI, 0.63–1.17), and 8.7% (HR, 0.76; 95% CI, 0.55–1.05), respectively, and the increase in T4N2b patients was 9.8%. These outcomes suggested that SOX adjuvant treatment could be feasible for the patients with colorectal cancer with IIIC or T4 or N2b patients.

There was difference in duration and dosage of oxaliplatin between 6 months and 3 months arms. However, the aim of our study is basically an exploratory examination of optimal therapeutic dosage and duration in each arm, although it was not a comparative study between the two arms.

The reason why the oxaliplatin dosage in 6 months arm was 100 mg /m^2^ was because it was the same dose as the ACTS‐CC 02 trial, which was the adjuvant treatment of colon cancer, and the G‐SOX regimen for the treatment of advanced gastric cancer.^16^, ^23^ And the reason why the dose in the 3 months arm was 130 mg /m^2^ was because it was the same dose as first‐line regimen for metastatic colorectal cancer,^13^, ^14^ and because we expected that shortening the duration to 3 months would improve oxaliplatin resistance even at 130 mg/m^2^. We showed that 130 mg/m^2^ dose of oxaliplatin was acceptable and feasible for 3 months in short‐term outcomes.

The duration of adjuvant treatment is as follows. In our study, the 3‐year DFS in 3 months arm was 76.9% compared to 75.0% in the 6 months arm, although our study wasn't a comparative trial between 6 months and 3 months arm. Moreover, this study has failed to show that primary endpoint of 3‐year DFS was superior to 72% for 3 months or 6 months SOX adjuvant therapy. The ACHIEVE trial showed the 3‐year DFS for 3 months and 6 months in CAPOX arm; a 3‐year DFS rate of 81.4% (95% CI, 77.6–84.6) for 3 months versus 79.7% (95% CI, 75.8–83.1) for 6 months treatment (HR, 0.90; 95% CI, 0.68–1.20).^3^ The dosage of oxaliplatin was 130 mg/m^2^ in both 3 and 6 months groups of the ACHIEVE trial, and also in 3 months arm of our study. It was 100 mg/m^2^ in 6 months arm of our study. The treatment completion rates were 86% in 3 months and 60% in 6 months arm in the ACHIEVE trial compared to 86.1% and 73.7% in our study.^24^ The RDI of oxaliplatin / capecitabin (ACHIEVE trial) or S‐1 (our trial) was 92%/88% in 3 months arm and 78%/78% in 6 months arm compared to 82.6%/85% and 76.9%/77.4% in our study.^24^ The complication rate was higher in 6 months arm of our study than ACHIEVE trial and the RDI of oxaliplatin was lower in 3 months arm of our study than ACHIEVE study. As a result, the 3‐year DFS in our study was a little lower in not only 6 months arm but 3 months arms compared to ACHIEVE trials. This could be due to the differences between the two oral fluoropyrimidines, S‐1 and capecitabine, not just the RDI issue, or because the SOX regimen was not suitable for adjuvant settings.

One of the merits of the shortening of the duration of adjuvant therapy is reducing the peripheral neuropathy for the patients. In ACHIEVE study, the 3 months oxaliplatin‐based adjuvant therapy resulted in reduction peripheral neuropathy by 1/2 or 1/3 compared to 6 months therapy.^3^ In our study there was statically no difference between 6 months and 3 months arm until 12 months of the end of the treatment. After 3 months of the end of the treatment, the PNQ score of the sensory and motor was slightly higher in 6 months compared to 3 months arm. However, the difference of the grade was gradually decreased and no difference after 12 months of the finish the treatment. There were some reasons of no difference between two arms. One was that the completion rate of the treatment was about 12% higher in 3 months arm than in 6 months arm and RDI of SOX treatment was also significantly higher in 3 months than 6 months arm. The strength of the peripheral neuropathy depends on the amount of oxaliplatin. And in cohort study showed that the risk factor of oxaliplatin‐induced CIPN post treatment were low pretreatment hemoglobin, higher body mass index, older age, and female sex.^25^ There was no difference of hemoglobin, age, body mass index, and the being female between two arms in our study.

### 4.1 Limitation

Our study had some limitations that should be considered. First, all participant patients of our study were Japanese. And S‐1 is launched in 13 Asian countries (Japan, South Korea, China, Singapore, Taiwan, Thailand, Hong Kong, Malaysia, Myanmar, Laos, etc.) and 17 European countries (UK, Sweden, Germany, Austria, etc.). It has not been released in USA and some European countries. And it is only approved for gastric cancer in some of them.

Secondary, JCOG0910 study was at the prespecified second interim analysis after final accrual, the Data and Safety Monitoring Committee recommended early publication on September 24 because S‐1 could not show non‐inferiority compared with capecitabine for DFS, 2014. Our study was planned in January 2014 and had already started at that time.

Thirdly, the outcome of ACTS‐CC 02 was published in 2020. This outcome showed SOX adjuvant regimen wasn't superior to UFT/LV for the high‐risk stage III colon cancer. However, the final randomized cases were on November 17, 2017, in our study.

Finally, we tried to collect PNQ from participants after the protocol treatment. However, the PNQ collection rate was low and insufficient to accurately assess the extent of peripheral neuropathy.

## 5 CONCLUSIONS

The 80% confidence interval of 3‐year DFS was not superior to 72% in both 3 months and 6 months arms for the patients of stage III colon cancer, and primary endpoint was not achieved. However, the SOX regimens were safe in both arms in short outcomes. In conclusion, the SOX regimen was not feasible in long‐term outcomes.

## FUNDING INFORMATION

This works was funded by TAIHO PHARMACEUTICAL CO. Ltd., Japan under the research contract.

## CONFLICT OF INTEREST STATEMENT

The authors declare no conflicts of interest for this article. The authors, Jun Watanabe and Itaru Endo, are editorial members of *Annals of Gastroenterological Surgery*.

## ETHICAL APPROVAL

Approval of the research protocol: The protocol for this research project has been approved by a suitably constituted Ethics Committee of the Yokohama City University (Approval no. CRB18‐014) and it conforms to the provisions of the Declaration of Helsinki.

Informed consent: All written informed consent was obtained from the subjects.

Registry and the Registration No. of the study: UMIN000013825.

Animal Studies: N/A.
